# Assessing the safety and use of medicinal herbs during pregnancy: a cross-sectional study in São Paulo, Brazil

**DOI:** 10.3389/fphar.2023.1268185

**Published:** 2023-09-19

**Authors:** Hyea Bin Im, Ricardo Ghelman, Caio Fábio Schlechta Portella, Jung Hye Hwang, Dain Choi, Sangita Karki Kunwor, Sandra Dircinha Teixeira de Araújo Moraes, Dongwoon Han

**Affiliations:** ^1^ Department of Global Health and Development, Graduate School, Hanyang University, Seoul, Republic of Korea; ^2^ Institute of Health Services Management, Hanyang University, Seoul, Republic of Korea; ^3^ Brazilian Academic Consortium for Integrative Health, São Paulo, Brazil; ^4^ School of Medicine, University of São Paulo, São Paulo, Brazil; ^5^ Department of Obstetrics and Gynecology, College of Medicine, Hanyang University, Seoul, Republic of Korea; ^6^ Hospital e Maternidade Amador Aguiar, São Paulo, Brazil; ^7^ Department of Preventive Medicine, College of Medicine, Hanyang University, Seoul, Republic of Korea

**Keywords:** herbal medicine, safety classification, pregnant women, cross-sectional study, Brazil

## Abstract

**Background:** Despite the lack of evidence supporting the safety and clinical efficacy of herbal medicine (HM), its use among pregnant women continues to increase. Given the high prevalence of contraindicated herbs among the pregnant population in Brazil, it is crucial to examine the use of HM and evaluate its safety based on the current scientific literature to ensure that women are using HM appropriately.

**Methods:** A cross-sectional study was conducted from October 2022 to January 2023 at a public teaching hospital in São Paulo, Brazil. A total of 333 postpartum women in the postnatal wards and postnatal clinic were interviewed using a semi-structured questionnaire. The survey instrument consisted of 51 items covering the use of HM during pregnancy, sociodemographic and health-related characteristics, COVID-19 experiences, and pregnancy outcomes. For data analysis, chi-square and multivariate logistic regression were conducted using SPSS ver. 26.0.

**Results:** Approximately 20% of respondents reported using HM during their most recent pregnancy, with a higher use observed among women from ethnic minority groups and those with prior HM experience. Among the 20 medicinal herbs identified, 40% were found to be contraindicated or recommended for use with caution during pregnancy. However, only half of the women discussed their HM use with obstetric care providers.

**Conclusion:** This study emphasizes the continued public health concern regarding the use of contraindicated or potentially harmful HM among pregnant women in Brazil, highlighting the need for sustained efforts to reduce the risk of inappropriate HM use. By updating antenatal care guidelines based on the latest scientific evidence, healthcare providers can make informed clinical decisions and effectively monitor pregnant women’s HM use, ultimately promoting safer and more effective healthcare practices.

## 1 Introduction

Globally, complementary and alternative medicine (CAM) has become an integral part of healthcare practices for several decades, influenced by cultural traditions, historical values, and the growing demand for holistic approaches to healthcare (Hasan et al., 2009; Islahudin et al., 2017; James et al., 2018b). Recognizing the significance of CAM in promoting patient autonomy and patient-centered care, Brazil officially integrated CAM into its Unified Health System (SUS) through a national ordinance in 2006 (Series, 2008; Boccolini et al., 2022). According to the National Health Survey (PNS), approximately 5% of Brazilian adults use CAM, with a greater prevalence among women (Boccolini et al., 2022). Furthermore, 13%–30% of pregnant women in Brazil reported using medicinal herbs, indicating a significantly higher prevalence compared to the general population (Araújo et al., 2016; da Matta et al., 2021).

Interest in the use of herbal medicine (HM) among pregnant women persists (Abdollahi and Chareti, 2019; El Hajj and Holst, 2020; Jahan et al., 2022), as pregnant women seek ways to manage various health conditions during pregnancy while avoiding potential risks associated with synthetic medications (Nyeko et al., 2016; Ahmed et al., 2018; James et al., 2018a). However, despite their belief in HM as a safer alternative to conventional drugs, concerns have been raised regarding the safety and effectiveness of HM use during pregnancy (Marcus and Snodgrass, 2005; Kennedy et al., 2016; Ahmed et al., 2017; El Hajj and Holst, 2020). The use of HM is predominantly supported by anecdotal evidence, and many herbs lack scientific research to determine their safety during pregnancy (Frawley et al., 2014; Smeriglio et al., 2014). Moreover, even for the herbs that have been studied extensively, conflicting clinical recommendations exist due to varying opinions on consumption limits (Ahmed et al., 2017; Sarecka-Hujar and Szulc-Musioł, 2022). As a result, the discrepancies in available evidence and its application in healthcare pose health risks to pregnant women and the developing fetus, highlighting the need to address the knowledge gaps and promote evidence-based guidelines.

Similar concerns have emerged in Brazil, where certain herbs commonly used by Brazilian women have been identified as potentially harmful during pregnancy (Moreira et al., 2014; Araújo et al., 2016). For example, Brazilian women frequently consume *Peumus boldus* Mol., *Foeniculum vulgare* Mill., and *Cymbopogon citratus* Stapf., despite the teratogenic and abortifacient properties of these herbs (Araújo et al., 2016; Casagrande et al., 2023; Souza et al., 2023). Many women persist in using these contraindicated herbs, inadvertently endangering themselves and their babies due to simple ignorance (da Matta et al., 2021). Furthermore, previous safety evaluations of Brazilian herbs are primarily based on Resolution SES/RJ No. 1757, which was published in 2002 (Saúde, 2002; Araújo et al., 2016; da Matta et al., 2021). Considering the growing popularity of HM use among pregnant women in Brazil, it is crucial to investigate the use of HM in different regions of Brazil and assess the safety of commonly used herbs based on current scientific evidence. Therefore, this study aims to explore the use of HM among pregnant women in São Paulo, the most populous city in Brazil, and evaluate the safety profile of the identified herbs.

## 2 Materials and methods

### 2.1 Study setting and participants

A descriptive cross-sectional study was conducted to examine HM use during pregnancy in São Paulo, Brazil. The eligible participants included postpartum women between the ages of 18 and 49 years who were admitted to the postnatal wards or visited the postnatal clinic at Hospital e Maternidade Amador Aguiar, a public teaching hospital in São Paulo, Brazil. In addition, only those who had given birth within the last 6 weeks were invited to participate in the survey to minimize recall bias. Women with any type of disabilities, currently receiving treatment for a severe condition in a high dependency or intensive care unit, or those who did not consent to participate in the study were excluded from participation.

### 2.2 Study size

The required sample size was calculated using a formula based on the confidence interval (CI). In this equation, n represents the required sample size, Z_α/2_ is set at 1.96 for a confidence interval of 95%, d denotes the margin of error fixed at 0.05, p stands for the expected proportion of pregnant women’s HM use, as determined from previous literature (22.3%) (Kennedy et al., 2013; Araújo et al., 2016), and q signifies the proportion of women not using HM during pregnancy (1-p). To achieve adequate statistical power, a sample size of 266 was determined, and assuming a 30% non-response rate, a total of 340 survey questionnaires were distributed.

### 2.3 Survey instrument

The semi-structured questionnaire was first developed in English based on previous studies investigating HM use among pregnant women (Al-Ramahi et al., 2013; Araújo et al., 2016; Hwang et al., 2016; Ahmed et al., 2018; da Matta et al., 2021). In order to measure the content and face validity of the survey instrument, the questionnaire was reviewed by four experts (two maternal and child health experts in Korea, one pediatrician, and one gynecologist from Brazil) for clarity, relevance, appropriateness, adequacy, and organization of the questions. The questionnaire was then translated into Portuguese and subsequently back-translated into English to ensure linguistic validity. The questionnaire was pilot-tested on a sample of 10 participants to examine the length, clarity, and difficulty of the questions, and based on the results, a few items were modified into the final version.

The final version of the questionnaire consisted of four sections with 51 items with a combination of multiple-choice and open-ended questions. The first section included 19 questions on the health-related characteristics of participants, such as current health status measured by a 5-point Likert scale, smoking status, morbidities, medication history, number of antenatal care visits, satisfaction with the conventional medical care, perceived patient-doctor relationship, type of delivery, obstetric history, complications experienced during pregnancy, labor, and after birth, and experiences during the COVID-19 pandemic.

The second part of the survey contained 18 questions regarding HM use during pregnancy (i.e., history of HM use before pregnancy, use of HM during pregnancy, types of modalities used, indications for use, the reason for use and non-use, frequency of HM use, level of satisfaction, the experience of adverse effects, source of information, and physician-patient communication on HM use during antenatal consultations). To investigate the types of HM used, a list of frequently used herbs was presented to women for selection, and to account for any herbs not covered in the list, the interviewers explicitly asked participants to mention any additional herbs they might have used during pregnancy. Additionally, since certain herbal modalities are also frequently ingested as part of dietary habits, women were specifically instructed to select only those modalities used for medicinal purposes to ensure the selection of herbs with medicinal applications.

The third section included six questions on characteristics of the newborn baby (i.e., gestational age at birth, gender, weight, general health status, congenital malformations, and neonatal symptoms experienced such as breathing problems, newborn jaundice, tender abdomen, temperature problems, bowel problems, lethargy, cardiac symptoms, and convulsions). Lastly, the final section consisted of eight questions on respondents’ sociodemographic characteristics, such as age, area of residence, education level, employment status, ethnicity, household income, homeownership, and travel time to the nearest health facility.

### 2.4 Data collection

The study was approved by the Human Research Ethics Committee at Hospital e Maternidade Amador Aguiar in Brazil (CEP-HMAA No. 24/22). The interviewer-administered survey was conducted from October 2022 to January 2023, and one supervisor and two data collectors were recruited for data collection. All participation was voluntary, and before conducting the survey, informed consent approved by the institutional review board (IRB) was obtained. In addition, information on complications during labor and after childbirth, as well as the characteristics of the newborn baby were obtained with the help of hospital staff. A total of 340 women were invited to participate in the study, and 333 completed the survey (response rate: 97.9%).

### 2.5 Statistical analysis

In this study, the collected data from 333 participants were analyzed using Statistical Package for Social Sciences (SPSS) version 26.0. The descriptive statistics were used to examine the sociodemographic and HM use-related characteristics of the respondents. Pearson’s Chi-square test was performed to identify differences in sociodemographic and health-related characteristics between HM users and non-users. Furthermore, variables that were statistically significant in the Chi-square test were included in the regression for further analysis. The multivariate logistic regression analysis was conducted to determine factors associated with HM use during pregnancy, and a *p*-value of less than 0.05 was considered statistically significant for all analyses.

### 2.6 Safety classification of identified herbs

Following previous studies that determined the safety classifications of HM used among pregnant women, the HM identified in this study were also categorized into four groups: safe to use, should be used with caution, contraindicated, or insufficient evidence (Table 1) (Kennedy et al., 2016; Ahmed et al., 2017).

**TABLE 1:** Number of herbal medicines and users by safety categories.

Classification	Description	Number of herbs (%)	Reported number of uses (%)
Total		20 (100.0)	193 (100.0)
Safe to use	Evidence for the safety of herb in pregnancy is available	8 (40.0)	114 (59.1)
Should be used with caution	Caution must be taken when using this herb due to insufficient human evidence to support its safety or use of this herb can result in adverse effects	4 (20.0)	32 (16.6)
Contraindicated	The use of the herb during pregnancy demonstrated serious adverse effects on pregnant women or the fetus	4 (20.0)	18 (9.3)
Insufficient evidence	No evidence is available regarding the use of this herb in pregnancy	4 (20.0)	29 (15.0)

In order to evaluate the safety of each herb, a comprehensive review of the current scientific literature and relevant textbooks was conducted. The previous classification of HM used among pregnant women served as the primary reference source to determine the safety status, and the Botanical Safety Handbook (McGuffin, 1997) and Natural Standard Herb and Supplement Handbook (Basch and Ulbricht, 2005) were also reviewed to retrieve any relevant information regarding the safety of herbs. Furthermore, if an herb was not listed in the aforementioned sources, a Google Scholar search was performed using the following combination of search terms to identify the most recent evidence concerning its safety for pregnant women: ‘common herb name’ + ‘pregnancy’ or ‘formal scientific herb name’ + ‘pregnancy.’

## 3 Results

### 3.1 Sociodemographic characteristics of study participants

The sociodemographic characteristics of study respondents are shown in Table 2. The majority of respondents were aged between 21 and 30 years, had attained a high school education or above, and were currently unemployed. Approximately 19% of respondents used one or more types of HM during their most recent pregnancy. Significant differences between the HM users and non-users were observed in ethnicity (*p* = 0.017), home ownership status (*p* = 0.036), and travel time to the nearest health facility (*p* = 0.009).

**TABLE 2:** Socio-demographic characteristics of participants.

Variables	Total N = 333 (%)	HM users N = 63 (%)	Non-users N = 270 (%)	*p*-value
Age (mean 27.06 ± 6.55)				
20 years and below	56 (16.8)	11 (17.5)	45 (16.7)	0.961
21–30 years	185 (55.6)	34 (54.0)	151 (55.9)	
31 years and above	92 (27.6)	18 (28.6)	74 (27.4)	
Education level				
Elementary school or below	53 (15.9)	8 (12.7)	45 (16.7)	0.066
Middle school	57 (17.1)	17 (27.0)	40 (14.8)	
High school or above	223 (67.0)	38 (60.3)	185 (68.5)	
Employment status				
Yes	90 (27.0)	21 (33.3)	69 (25.6)	0.211
No	243 (73.0)	42 (66.7)	201 (74.4)	
Ethnicity				
Caucasian	89 (26.7)	21 (33.3)	68 (25.2)	0.017
Mulatto	166 (49.8)	24 (38.1)	142 (52.6)	
Black	59 (17.7)	10 (15.9)	49 (18.1)	
Others	19 (5.7)	8 (12.7)	11 (4.1)	
Homeownership				
Yes	73 (21.9)	20 (31.7)	53 (19.6)	0.036
No (rented)	260 (78.1)	43 (68.3)	217 (80.4)	
Travel time to the nearest health facility				
Less than 30 min	125 (37.5)	21 (33.3)	104 (38.5)	0.009
30 min to 1 h	97 (29.1)	28 (44.4)	69 (25.6)	
More than 1 h	111 (33.3)	14 (22.2)	97 (35.9)	

### 3.2 Medical characteristics of study participants

As shown in Table 3, most respondents had no prior history of gynecological conditions (80.5%) or complications during pregnancy (78.4%). However, a greater percentage of HM users encountered complications during or after delivery (*p* = 0.003). Furthermore, HM users had a higher prevalence of taking pain medications (*p* < 0.001), vitamins (*p* < 0.001), and other types of medications (*p* = 0.042) during their pregnancy compared to non-users.

**TABLE 3:** Medical characteristics of study participants.

Variables	Total N = 333 (%)	HM users N = 63 (%)	Non-users N = 270 (%)	*p*-value
Medications taken during pregnancy^a^ ^,^ ^b^				
Pain medicine	122 (36.6)	37 (58.7)	85 (31.5)	<0.001
Antibiotics	72 (21.6)	19 (30.2)	53 (19.6)	0.068
Folic acid	265 (79.6)	46 (73.0)	219 (81.1)	0.151
Vitamins	16 (4.8)	15 (23.8)	1 (0.4)	<0.001
Others	43 (12.9)	13 (20.6)	30 (11.1)	0.042
Previous experience of gynecological conditions^a^				
Yes	65 (19.5)	16 (25.4)	49 (18.1)	0.191
No	268 (80.5)	47 (74.6)	221 (81.9)	
Complications during pregnancy^a^				
Yes	72 (21.6)	17 (27.0)	55 (20.4)	0.251
No	261 (78.4)	46 (73.0)	215 (79.6)	
Complications during/after childbirth^a^				
Yes	58 (17.4)	19 (30.2)	39 (14.4)	0.003
No	275 (82.6)	44 (69.8)	231 (85.6)	
Experience of newborn problems^a^ ^,^ ^b^				
None	258 (77.5)	44 (69.8)	214 (79.3)	0.107
Breathing problems	20 (6.0)	6 (9.5)	14 (5.2)	0.234
Appearance (blue or pale)	5 (1.5)	4 (6.3)	1 (0.4)	0.005
Newborn jaundice (yellow skin, eyes)	21 (6.3)	10 (15.9)	11 (4.1)	0.001
Convulsions/unconsciousness	2 (0.6)	2 (3.2)	0 (0.0)	0.035
Excessively irritable and crying	2 (0.6)	2 (3.2)	0 (0.0)	0.035
Tender or tense abdomen	3 (0.9)	3 (4.8)	0 (0.0)	0.007
Any infections	4 (1.2)	3 (4.8)	1 (0.4)	0.022
Bowel problems (blood in the bowel, diarrhea)	1 (0.3)	1 (1.6)	0 (0.0)	0.189
Cardiac problems (abnormal heart rate or blood pressure)	5 (1.5)	3 (4.8)	2 (0.7)	0.049
Preterm birth	162 (48.6)	33 (52.4)	129 (47.8)	0.510
Prior use of HM				
Yes	50 (15.0)	31 (49.2)	19 (7.0)	<0.001
No	283 (85.0)	32 (50.8)	251 (93.0)	

^a^ Columns do not add up to 100% due to the selection of multiple answers.

^b^ A chi-square analysis was performed for each response item, treating each item as a separate group with two options (‘yes’ or ‘no').

Approximately 23% of respondents reported experiencing problems with their newborns after birth. A significantly higher proportion of newborns with blue or pale appearance (*p* = 0.005), newborn jaundice (*p* = 0.001), convulsions (*p* = 0.035), excessive irritability or crying (*p* = 0.035), tender or tense abdomen (*p* = 0.007), any type of infections (*p* = 0.022), and cardiac problems (*p* = 0.049) were found in Herbal Medicine user group than the non-users. Lastly, a larger proportion of HM users had previous experience with HM use compared to non-users (Table 3).

### 3.3 Experiences during the COVID-19 pandemic

Table 4 presents respondents’ experiences during the COVID-19 pandemic. HM users reported significantly higher incidences of emotional and physical challenges compared to non-users during the COVID-19 pandemic. These challenges include fear of the infection (*p* = 0.019), emotional distress (*p* = 0.012), difficulty visiting health facilities or services (*p* = 0.006), feeling confined at home (*p* = 0.028), financial difficulties or job loss (*p* = 0.040), maintaining relationships with family and friends (*p* = 0.003), and experiencing loneliness (*p* = 0.003). Furthermore, a greater proportion of HM users perceived their health status to be worse during the COVID-19 pandemic (*p* < 0.001) and were diagnosed with COVID-19 during their pregnancy (*p* = 0.010).

**TABLE 4:** Experiences during the COVID-19 pandemic.

Variables	Total N = 333 (%)	HM users N = 63 (%)	Non-users N = 270 (%)	*p-*value
Challenges and struggles faced during the COVID-19 pandemic^a^ ^,^ ^b^				
None	145 (43.5)	14 (22.2)	131 (48.5)	<0.001
Fear of getting infected and infecting the baby	136 (40.8)	34 (54.0)	102 (37.8)	0.019
Feeling more stressed out	72 (21.6)	21 (33.3)	51 (18.9)	0.012
Difficulty visiting health facilities or accessing health services	56 (16.8)	18 (28.6)	38 (14.1)	0.006
Feeling confined at home	54 (16.2)	16 (25.4)	38 (14.1)	0.028
Financial difficulties/job loss	60 (18.0)	17 (27.0)	43 (15.9)	0.040
Difficulty sleeping	36 (10.8)	11 (17.5)	25 (9.3)	0.059
Maintaining relationships with family and friends	46 (13.8)	16 (25.4)	30 (11.1)	0.003
Loneliness	24 (7.2)	10 (15.9)	14 (5.2)	0.003
Health status compared to before the pandemic				
Worse	23 (6.9)	11 (17.5)	12 (4.4)	<0.001
About the same	280 (84.1)	44 (69.8)	236 (87.4)	
Better	30 (9.0)	8 (12.7)	22 (8.1)	
Diagnosed with COVID-19				
Yes	89 (26.7)	25 (39.7)	64 (23.7)	0.010
No	244 (73.3)	38 (60.3)	206 (76.3)	

^a^ Columns do not add up to 100% due to the selection of multiple answers.

^b^ A chi-square analysis was performed for each response item, treating each item as a separate group with two options (‘yes’ or ‘no').

### 3.4 Types of HM used by its safety classification

Among pregnant women in Brazil, the use of 20 different herbs was identified (Table 5). Eight out of 20 herbs were found to be contraindicated or recommended for use with caution during pregnancy. However, the majority of HM users consumed HM that was considered safe during pregnancy, such as *Matricaria recutita* L. (52.4%) and *Allium sativum* L. (39.7%). Among potentially harmful HM, the prevalence of *Citrus aurantifolia* Swingle consumption was the highest (41.3%).

**TABLE 5:** Types of HM used during pregnancy and indication for use.

Types of HM		n (%)	Study subjects in referenced studies	Safety documentation
Modalities used^a^ (*n* = 63)				
Safe to use during pregnancy				
Chamomile	*Matricaria recutita* L.	33 (52.4)	Human	Its consumption as tea is safe in moderate amounts Kennedy et al. (2016); Ahmed et al. (2017), but excessive use should be avoided due to its potential effect as a uterine stimulant and the association with premature constriction of fetal ductus arteriosus Basch and Ulbricht (2005); Sridharan et al. (2009).
Garlic	*Allium sativum* L.	25 (39.7)	Human	Its use in pregnancy is safe with no adverse effects when consumed in the typical amounts found in food Aalami-Harandi et al. (2015); Ahmed et al. (2017). However, it should not be consumed in large quantities due to an increased risk of bleeding and gastrointestinal discomfort Basch and Ulbricht (2005).
Lemon	*Citrus limon* (L.) Osbeck.	18 (28.6)	Human	Clinical evidence involving human subjects has not reported any harmful effects of lemon use during pregnancy McGuffin (1997); Safajou et al. (2014).
Olive oil	*Olea europaea* L.	13 (20.6)	Human	Studies conducted on pregnant women have not shown any adverse effects of olive or olive oil use Taavoni et al. (2011); Kennedy et al. (2016); Ahmed et al. (2017); Gomez Ribot et al. (2020).
Green tea	*Camellia sinensis* L. Kuntze	8 (12.7)	Human	Its consumption as tea is generally safe, but large amounts should be avoided due to its caffeine content and potential teratogenic effects Kennedy et al. (2016); Ahmed et al. (2017).
Ginger	*Zingiber officinale* Rosc.	7 (11.1)	Human	Studies have shown no harmful effects on both mother and fetus when it is used in moderate amounts Heitmann et al. (2013); Viljoen et al. (2014); Ahmed et al. (2017), but it should not be used in high doses due to potential toxicity Basch and Ulbricht (2005).
Peppermint	*Mentha piperita* L.	7 (11.1)	Human	Its use as tea has not been associated with any adverse effects Kennedy et al. (2016); Ahmed et al. (2017), yet it should not be used in large amounts due to its emmenagogue and teratogenic effects Saúde (2002); Basch and Ulbricht (2005); Araújo et al. (2016).
Eucalyptus	*Eucalyptus globulus* Labill.	3 (4.8)	Human	Topical application of its oil is considered safe during pregnancy McGuffin (1997); Kennedy et al. (2016); Ahmed et al. (2017)), but oral ingestion of eucalyptus oil should be avoided due to potential harmful effects, such as dizziness, drowsiness, gastrointestinal problems, and even coma Basch and Ulbricht (2005).
Should be used with caution				
Lime	*Citrus aurantifolia* Swingle	26 (41.3)	Animal	An animal study has shown potential abortifacient effects Bakare et al. (2012), but limited clinical evidence is available to determine the safety of its use in human pregnancy McGuffin (1997); Kennedy et al. (2016).
Fennel	*Foeniculum vulgare* Mill	4 (6.3)	Animal	Its consumption as tea is safe during pregnancy, yet its oil and alcohol extracts should be avoided due to potential adverse effects McGuffin (1997); Saúde (2002); Araújo et al. (2016); Kennedy et al. (2016); Mahboubi (2019); Bebitoglu (2020). Furthermore, regular consumption of fennel was significantly associated with reduced gestational age Trabace et al. (2015).
Valerian	*Valeriana officinalis* L.	1 (1.6)	Animal	Not recommended for use during pregnancy due to a lack of safety data in human pregnancy Basch and Ulbricht (2005); Kennedy et al. (2016). Also, animal studies have reported inconclusive findings regarding its effect on fetal development Yao et al. (2007); Mahmoudian et al. (2012).
Evening primrose	*Oenothera biennis* L.	1 (1.6)	Human	A small study found a potential association between its use and an increased risk of pregnancy complications Dove and Johnson (1999). Insufficient evidence is available to establish its safety during human pregnancy Basch and Ulbricht (2005); Kennedy et al. (2016)
Contraindicated				
Cinnamon	*Cinnamomum verum* J. Presl.	8 (12.7)	Animal	Consumption of its oil has been linked to embryo loss and an increase in fetal malformation; therefore, it should only be used in amounts commonly found in food McGuffin (1997); Kennedy et al. (2016); Ahmed et al. (2017).
Lemongrass	*Cymbopogon citratus* Stapf.	7 (11.1)	Animal	Its use in high doses has shown developmental toxicity and abortifacient effects McGuffin (1997); Saúde (2002); Ekpenyong et al. (2014).
Thyme	*Thymus vulgaris* L.	2 (3.2)	Animal	Excessive consumption should be avoided due to its potential effects on fetal growth and abortifacient properties McGuffin (1997); Kennedy et al. (2016); Ahmed et al. (2017); Tafesh et al. (2021).
Boldo	*Peumus boldus* Mol.	1 (1.6)	Animal	Animal evidence has suggested potential fetotoxic and teratogenic effects McGuffin (1997); Saúde (2002); Kennedy et al. (2016).
Insufficient safety evidence				
Passion fruit	*Passiflora edulis* Sims.	24 (38.1)	Human	Although no side effects have been reported from the topical use of its cream, limited scientific evidence is available regarding its use and safety among pregnant women Aryunisari et al. (2021).
Horse-chestnut	*Aesculus hippocastanum* L.	2 (3.2)	Human	No serious adverse effects were reported from a small study involving pregnant women, but insufficient scientific data is available regarding its use and safety during pregnancy Basch and Ulbricht (2005).
Lemon balm	*Melissa officinalis* L.	2 (3.2)	N/A	Limited scientific evidence is available to establish the safety of its use during pregnancy McGuffin (1997); Kennedy et al. (2016).
Rosehip	*Rosa canina* L.	1 (1.6)	N/A	Its use in pregnancy is not recommended due to the lack of evidence regarding its safety Tapasvi (2023).

^a^ Columns do not add up to 100% due to the selection of multiple answers.

### 3.5 Patterns of HM use during pregnancy

While women turned to HM for various reasons, the most reported indications for HM use during pregnancy were nausea and vomiting (58.7%), followed by heartburn and indigestion (47.6%), as well as anxiety and stress (31.7%). The most commonly reported source of information on HM were family and friends (61.9%), and 49.2% of women did not disclose their HM use to the healthcare providers, primarily due to the lack of physician inquiry and no specific reason. The main reasons for HM use were beliefs in its effectiveness (58.7%) and safety (20.6%), along with family traditions and cultural influences (20.6%). However, the majority of non-users decided not to use HM because they were satisfied with conventional medicine (Table 6).

**TABLE 6:** Patterns of HM use during pregnancy.

Variables	Frequency	Percentage
Indications for use^a^ (n = 63)		
Nausea/vomiting	37	58.7
Heartburn/indigestion	30	47.6
Anxiety/stress	20	31.7
Cough/cold/flu	17	27.0
Back pain/joint pain	16	25.4
Urinary tract infection	14	22.2
Fatigue	11	17.5
Stomach problems/constipation	6	9.5
Protect against COVID-19	5	7.9
Prepare for easy labor	4	6.3
Improve general health status	3	4.8
Fetal health promotion	2	3.2
Others	9	14.3
Source of recommendation^a^ (*n* = 63)		
Family/friends/neighbor	39	61.9
Doctor	15	23.8
Midwife or health worker	9	14.3
TV/radio/internet	2	3.2
Others	11	17.5
Reason for HM use^a^ (*n* = 63)		
I believe it’s effective	37	58.7
I believe it’s safe	13	20.6
Family, tradition, or culture	13	20.6
It was recommended by the doctor/midwife	11	17.5
It’s cheap and accessible	9	14.3
Others	19	30.2
Reason for non-use^a^ (*n* = 270)		
I am satisfied with conventional medicine	191	70.7
It is not effective	13	4.8
It is expensive and difficult to get	6	2.2
My family did not let me use	6	2.2
It is not safe	4	1.5
My doctor/nurse did not let me use	2	0.7
Others	58	21.5
Disclosure of HM use (*n* = 63)		
Yes	32	50.8
No	31	49.2
Reason for non-disclosure^a^ (*n* = 31)		
The doctor did not ask	11	35.5
No specific reason	11	35.5
Did not think physicians needed to know	7	22.6
Should have informed but forgot	2	6.5
Did not have time to discuss with the doctor	1	3.2
Intention to disclose HM use if doctor inquired (*n* = 63)		
Yes	55	87.3
No	8	12.7

^a^ Columns do not add up to 100% due to the selection of multiple answers.

### 3.6 Potential predictors of HM use during pregnancy

The results of the multivariate logistic regression analysis revealed that belonging to a minor ethnicity (OR: 5.536; CI: 1.402–21.862) and having previous experience with HM use (OR: 16.216; CI: 7.244–36.300) were identified as potential predictors of HM use during pregnancy (Table 7).

**TABLE 7:** Potential predictors of HM use during pregnancy.

Variables	OR	95% CI	*p*-value
Ethnicity			
Caucasian	1	Ref.	
Mulatto	1.410	0.497–3.998	0.519
Black	0.888	0.327–2.411	0.816
Others	5.536	1.402–21.862	0.015
Homeownership			
No (rented)	1	Ref.	
Yes	1.714	0.798–3.681	0.167
Travel time to the nearest health facility			
Less than 30 min	1	Ref.	
30 min to 1 h	2.578	0.990–6.708	0.052
More than 1 h	1.367	0.544–3.434	0.506
Medication intake			
No	1	Ref.	
Yes	1.939	0.821–4.579	0.131
Complications during/after childbirth			
No	1	Ref.	
Yes	1.061	0.467–2.410	0.888
Prior use of HM			
No	1	Ref.	
Yes	16.216	7.244–36.300	<0.001
Challenges and struggles faced during the COVID-19 pandemic			
Yes	1	Ref.	
No	0.731	0.330–1.620	0.440
Health status compared to before the pandemic			
Worse	1	Ref.	
About the same	3.063	0.756–12.414	0.117
Better	0.627	0.212–1.851	0.398
Diagnosed with COVID-19			
No	1	Ref.	
Yes	1.040	0.506–2.141	0.914

## 4 Discussion

This cross-sectional study investigated the use of HM among pregnant women in São Paulo, Brazil, and our findings revealed that approximately 20% of Brazilian women used HM during pregnancy. The observed prevalence was lower than the findings reported in a previous study conducted in the northeastern part of Brazil, and this discrepancy can be attributed to variations in sample sizes of the studies and cultural diversity in the studied regions (Araújo et al., 2016). However, when comparing the prevalence with that of Brazilian adults, as reported in the National Health Survey, pregnant women demonstrated a higher prevalence of complementary medicine use (de Moraes Mello Boccolini and Siqueira Boccolini, 2020; Boccolini et al., 2022). This suggests that pregnant women are more likely to explore natural treatment options for alleviating physical discomforts, as the women are trying to protect their developing fetus from any teratogenic and fetotoxic effects of conventional medications (John and Shantakumari, 2015; Illamola et al., 2020). Similarly, a significant number of women in our study reported using HM during pregnancy based on their perceived effectiveness and safety (Borrelli et al., 2007). These findings provide insights into the beliefs and perceptions of Brazilian pregnant women regarding HM as a safe and viable treatment option.

In line with previous findings, minor ethnicity and previous experience of HM use were found as potential predictors of HM use during pregnancy (Kalder et al., 2011; Hall and Jolly, 2014; Pallivalappila et al., 2014; El Hajj et al., 2020). Many minor ethnic groups have a longstanding history of traditional medicine use, and the use of HM is often rooted in traditional health practices (Ray et al., 2018; Agu et al., 2019). Consequently, individuals who belong to minor ethnic groups and have a cultural background in the use of HM may have a greater tendency to use such remedies. Furthermore, having prior experience of HM use and being familiar with HM could influence the women to be more comfortable using HM; therefore, women who have previously used HM are more likely to continue its use during pregnancy (Pallivalappila et al., 2014; El Hajj et al., 2020; Ahmed et al., 2022).

Our study identified the use of 20 different types of HM during pregnancy, and 40% of these herbs were found to be contraindicated or recommended with caution. Among the herbs that should be used with caution, the prevalence of *C. aurantifolia* intake was most prevalent. *C. aurantifolia* is commonly used as both a food product and a phytomedicine, due to its diverse range of beneficial properties, such as antibacterial, anticancer, and anti-inflammatory effects (Narang and Jiraungkoorskul, 2016; Lee et al., 2018; Shafreen et al., 2018; Indriyani et al., 2023). Also, it is a popular home remedy among pregnant women for the treatment of morning sickness and urinary tract infections (Rahmawati, 2018; Ramadhani et al., 2020). However, an animal study found an abortifacient effect of lime juice, raising concerns about its safety during pregnancy (Bakare et al., 2012). Furthermore, excessive consumption of citrus fruits can lead to heartburn and indigestion, posing additional risks (Nundy, 2016; Hoffmann, 2017). Furthermore, using its essential oil at high concentration may result in necrosis and trigger an inflammatory response (Brah et al., 2023). Previous reviews have also advised against the use of *C. aurantifolia* due to the lack of sufficient clinical evidence regarding its safety (Kennedy et al., 2016; Ramadhani et al., 2020). Therefore, despite its widespread use and perceived safety, the current scientific evidence suggests that the therapeutic use of *C. aurantifolia* during pregnancy should be approached with caution.

Moreover, even herbs that are considered safe can have adverse effects if it is consumed in amounts more than typically found in food. For example, *M. recutita* is commonly used among pregnant women for flu, gastrointestinal irritation, and anxiety management (Ahmed et al., 2017; Bebitoglu, 2020; El Mihyaoui et al., 2022). Despite being considered safe for pregnancy (Kennedy et al., 2016; Ahmed et al., 2017; Al-Snafi and Hasham, 2023), prolonged administration of *M. recutita* was associated with a greater risk of preterm labor or miscarriage (Cuzzolin et al., 2010; Ahmed et al., 2017; Balbontín et al., 2019; Bebitoglu, 2020). Moreover, the Brazilian Resolution - SES/RJ No. 1757 categorizes *M. recutita* as contraindicated due to its emmenagogue properties (Saúde, 2002; Araújo et al., 2016). Similarly, *A. sativum*, another widely used HM among pregnant women in Brazil, has been identified as safe during pregnancy with no major adverse effects (Kennedy et al., 2016; Ahmed et al., 2017; Sarecka-Hujar and Szulc-Musioł, 2022; Sahidur et al., 2023). Nevertheless, consuming excessive amounts of *A. sativum* can result in gastrointestinal discomfort, and when used concomitantly with anticoagulants or antiretroviral drugs, it may cause drug-drug interactions (Borrelli et al., 2007; Kaur et al., 2013; Okoro et al., 2023). Considering that many herbs still lack comprehensive scientific investigations to definitively establish their safety, further clinical research is necessary to evaluate their potential toxicity during pregnancy. Moreover, maintaining active pharmacovigilance reporting is crucial to systematically record any adverse events that may arise.

In addition to the concerns regarding the lack of sufficient clinical evidence supporting the safety of HM, there are concerns related to the quality control of herbal products available in the market (Zhang et al., 2012; Okem et al., 2014). Even though some HM possess potent pharmacological properties, they are often classified as food or food additives and are not strictly regulated as pharmaceutical products (Heinrich, 2015). Consequently, there are potential risks associated with the quality of herbal products in the market, such as heavy metal contamination, inadequate supply chain management, and difficulties in controlling the potency of HM (Fong, 2002; Zhang et al., 2012; Okem et al., 2014; Luo et al., 2020). Consumption of contaminated or adulterated HM can have detrimental effects on health (Ezeabara et al., 2014). Therefore, it is crucial for pregnant women to exercise caution when using HM and consult their obstetric care providers before use.

Nevertheless, consistent with previous research, a significant number of pregnant women in our study obtained information about HM from family and friends, rather than healthcare professionals (Araújo et al., 2016; Ahmed et al., 2020; Eid and Jaradat, 2020; Ahmed et al., 2022). In addition, almost half of the women chose not to disclose their HM use to the obstetric care providers, either due to a lack of inquiry from the healthcare providers or for no specific reason. Such reliance on informal sources of information and the hesitancy to disclose HM use highlight the need for improved communication between pregnant women and obstetric care providers regarding HM. Furthermore, considering most women were willing to discuss HM use if the doctors initiated the conversation, obstetric care providers should play a proactive role in providing appropriate guidance and feedback regarding the use of HM during antenatal care consultations. Through these efforts, healthcare providers can effectively contribute to preventing potential health risks associated with the inappropriate use of HM during pregnancy by helping pregnant women make informed decisions and safer healthcare choices (Ahmed et al., 2020).

The current study has several limitations that should be considered when interpreting the results. Firstly, the generalizability of the study findings may be limited as the survey was conducted retrospectively at a single hospital in São Paulo. However, the data was collected from one of the public teaching hospitals that handle a substantial number of deliveries annually (>4,000 deliveries). In addition, to minimize the potential recall bias of the survey data, information was collected from postpartum women admitted to the maternal wards before discharge and those who had given birth within the last 6 weeks. Furthermore, due to the low frequency of delivery complications and congenital anomalies within our sample of 333 participants, we were unable to establish a significant association between HM use and pregnancy outcomes. This warrants further study with a more extensive and diverse sample size to draw more conclusive findings. Lastly, due to the cross-sectional study design, we could only establish associations between the variables and could not determine causality. Despite these limitations, our study provides valuable insights into HM use among pregnant women in Brazil based on current available evidence. In addition, our findings highlight the importance of developing reliable and evidence-based clinical guidelines to manage inappropriate HM use during pregnancy.

## 5 Conclusion

This study revealed that driven by misconceptions about the safety and effectiveness of HM, pregnant women in Brazil unknowingly jeopardize their own health and that of the newborns by using herbs that are contraindicated or potentially harmful during pregnancy. Updating antenatal care guidelines based on the latest scientific evidence and fostering transparent communication on HM use during consultations can help obstetric care providers make better clinical decisions and effectively monitor the health of both pregnant women and their babies. Lastly, sustained efforts must be taken to generate scientific evidence concerning the safety and clinical efficacy of commonly used HM, providing the foundation for informed medical practices and ultimately contributing to improved maternal and neonatal outcomes.

## Data Availability

The raw data supporting the conclusion of this article will be made available by the authors, without undue reservation.

## Abbreviations

HM, herbal medicine.
